# Derivation and validation of a prediction score for acute kidney injury secondary to acute myocardial infarction in Chinese patients

**DOI:** 10.1186/s12882-019-1379-x

**Published:** 2019-05-30

**Authors:** Feng-bo Xu, Hong Cheng, Tong Yue, Nan Ye, He-jia Zhang, Yi-pu Chen

**Affiliations:** Department of Nephrology, Beijing Anzhen Hospital, Capital Medical University, Beijing, People’s Republic of China

**Keywords:** Acute kidney injury, Acute myocardial infarction, Prediction score

## Abstract

**Background:**

Acute kidney injury (AKI) is a major complication of acute myocardial infarction(AMI), which can significantly increase mortality. This study is to analyze the related risk factors and establish a prediction score of acute kidney injury in order to take early measurement for prevention.

**Methods:**

The medical records of 6014 hospitalized patients with AMI in Beijing Anzhen Hospital from January 2010 to December 2016 were retrospectively analyzed. These patients were randomly assigned into two cohorts: one was for the derivation of prediction score (*n* = 4252) and another for validation (*n* = 1762). The criterion for AKI was defined as an increase in serum creatinine of ≥ 0.3 mg/dL or ≥ 50% from baseline within 48 h. On the basis of odds ratio obtained from multivariate logistic regression analysis, a prediction score of acute kidney injury after AMI was built up.

**Results:**

In this prediction score, risk score 1 point included hypertension history, heart rate > 100 bpm on admission, peak serum troponin I ≥ 100 μg/L, and time from admission to coronary reperfusion > 120 min; risks score 2 points included Killip classification ≥ class 3 on admission; and maximum dosage of intravenous furosemide ≥ 60 mg/d; risks score 3 points only included shock during hospitalization. In addition, when baseline estimated glomerular filtration rate (eGFR) was less than 90 ml/min·1.73 m^2^, every 10 ml/min·1.73 m^2^ reduction of eGFR increased risk score 1 point. Youden index showed that the best cut-off value for prediction of AKI was 3 points with a sensitivity of 71.1% and specificity 74.2%. The datasets of derivation and validation both displayed adequate discrimination (an area under the ROC curve, 0.79 and 0.81, respectively) and satisfactory calibration (Hosmer–Lemeshow statistic test, *P* = 0.63 and *P* = 0.60, respectively).

**Conclusions:**

In conclusion, a prediction score for AKI secondary to AMI in Chinese patients was established, which may help to prevent AKI early.

## Background

Acute kidney injury (AKI) has been reported to be a frequent complication of acute myocardial infarction (AMI) which is known to be associated with adverse outcomes [1]. The incidence of AKI in patients with AMI was 8.7 to 36.6% in the past 10 years [1–8]. AKI is associated with high mortality and also predicts the future risk for end-stage renal disease [8–11].

The mechanisms causing AKI secondary to AMI are multifactorial [12]. The key mechanisms in AKI pathogenesis including systemic and renal hemodynamic changes secondary to impaired cardiac output and increased venous congestion. Moreover, an imbalance of endogenous vasodilating and vasoconstrictive factors appears to be involved. A burst of immunological and inflammatory activation were the potential causes of further renal injury [12]. Several studies proposed certain risk factors for AKI secondary to AMI, including advanced age [6, 13, 14], admission hyperglycemia [15, 16], impaired renal function at presentation [5, 6, 13], and prolonged duration to coronary reperfusion [17]. There were some prediction scores of AKI after the percutaneous coronary intervention (PCI) for AMI [18–21]. However, only few studies have developed prediction scores including all the AMI patients which undergoing PCI or not [22, 23]. In 2012, Queiroz et al. created a prediction score for AKI secondary to AMI [22]. Nonetheless, the sample size was small (406 patients) and employed only for the clinical manifestation of ST-segment elevation myocardial infarction (STEMI) in the emergency department, thereby exhibiting some limitations. Recently, Abusaoda et al. developed a novel score to predict the risk of AKI secondary to AMI [23]. The study included a total of 1107 patients and the area under the receiver operating characteristic (ROC) curve (AUC) was 0.76. The above two prediction scores were primarily screened for those with AKI risk factors involve just at admission. The current study included patients with STEMI and non-STEMI, which undergoing PCI or not. This study analyzed the risk factors of AKI on admission as well as some potential risk factors of AKI also involve in the duration of hospital stay, and the prediction score was established with all these presentations. Therefore our prediction score showed adequate discrimination and good calibration, which could be used to screen the high-risk patients for AKI secondary to AMI more comprehensively and to help clinicians taking better preventive interventions.

## Methods

### Study design

We consecutively enrolled patients with AMI from Beijing Anzhen Hospital, one of the biggest cardiology center in China, from January 1, 2010 to December 31, 2016. All patients presented a primary diagnosis of AMI (STEMI or non-STEMI), and admitted to hospital within 24 h of onset of an ischemic event. The diagnosis of AMI was established by a typical history of chest pain, diagnostic electrocardiographic changes, and a successive elevation of serum cardiac biomarkers [24]. Exclusion criteria were (1) length of hospital stay< 2 days, (2) lacking sufficient inspection of serum creatinine (SCr), (3) pre-existing end stage renal disease requiring dialysis. The final analytic data from 6014 patients were included in our study. These patients were randomly assigned into two groups. The first group, comprising 70% of the patients (*n* = 4252), was used to derive the prediction score and the other group, consisting of the remaining 30% (*n* = 1762) patients, was used to validate the prediction score.

The definition of AKI was based on the change of measurements with SCr on admission using the AKI Network (AKIN) criteria. It was defined as an absolute increase in SCr levels of ≥0.3 mg/dL (26.4 μmol/L) or as a percent increase in SCr of ≥50% from baseline within 48 h. Moreover, AKI was classified into 3 stages based on an increase of 50–100% in baseline SCr (stage 1), 100–200% (stage 2), or > 300% or an increment of 0.5 mg/dL (44.2 μmol/L) if the baseline SCr was > 4.0 mg/dL (353.6 μmol/L) (stage 3) [25]. Due to the retrospective nature of the study, urine output in most patients was not monitored, and related data could not be obtained, and hence, this study did not consider the urine output standard.

Data on the following variables that might influence AKI development were collected: demographic data, previous history, clinical data, laboratory data, echocardiography data, and in-hospital treatment. Baseline SCr was defined as the value measured on admission. The baseline estimated glomerular filtration rate (eGFR) was calculated using the Modification of Diet in Renal Diseases (MDRD) equation for Chinese patients: eGFR (mL/ min  ⋅ 1.73m^2^) = 175 x SCr (mg/dL)^‐1.234^x Age^‐0.179^(× 0.79 for women) [26]. The dosage of loop diuretics was expressed as furosemide equivalents (1 mg bumetanide ≈ 20 mg torsemide≈ 40 mg furosemide).

A standardized data abstraction form was designed for data collection. The hospital records were abstracted by the trained medical record technicians. The strategies to decrease abstraction errors and variability included training sessions and detailed data definition.

### Statistical analysis

The continuous variables with normal distribution were presented as mean ± standard deviation, and *t*-test was used for univariate comparison. On the other hand, those with non-normal distribution were represented as median and interquartile range, and Wilcoxon–Mann–Whitney test was used for univariate comparison. The categorical variables were reported as percentages, and the chi-square test was used for univariate comparison. List wise deletion was used for missing data. The variables with *P* ≤ 0.05 in univariate comparison were included in the multivariate logistic regression analysis. Based on the odds ratio (OR) in the final multivariate logistic regression model, the risk factors for AKI were assigned weighted integers, and the prediction score was created. Discrimination of the prediction score was assessed using the ROC curve. Calibration was assessed using the Hosmer–Lemeshow goodness-of-fit test and satisfied when *P* value was > 0.05. The comparison of AUC between Abusaoda’s prediction score and our prediction score was done using the test proposed by DeLong et al. [27].

SPSS software version 17.0 (IBM Inc., Armonk, NY, USA) was used to analyze the data. For all analyses, *P* < 0.05 was considered as statistically significant.

## Results

### Incidence of AKI and in-hospital outcome

A total data from 6014 AMI patients, with mean age 58.00 ± 11.74 years, were recruited; and 80.5% of the patients consisted of males. AKI occurred in 675 patients (11.2%) including 9.5% stage 1, 1.1% stage 2, and 0.6% stage 3. The mortality rate was 10.1% in patients who developed AKI and 1.6% in those without AKI (*P <* 0.05). The mortality rate of the former was 6.31-fold higher than that of the latter. Moreover, the length of hospital stay in patients with AKI significantly prolonged with a median of 9 days (interquartile range, 6–14 days) as compared to 7 days (interquartile range, 5–9 days) in the patients without AKI (*P <* 0.05).

### Baseline characteristics and univariate analysis

The demographic data, previous history, clinical data, laboratory data, echocardiography data and in-hospital treatment of the patients in derivation cohort are shown in Table 1. The univariate comparison of potential predictor variables between AKI and non-AKI patients in the derivation cohort is also shown in Table 1. A total of 42 variables with *P*<0.05 in Table 1 were involved in the multivariate logistic regression model, but the data regarding N-terminal pro-B-type natriuretic peptide, glycosylated hemoglobin and echocardiographic parameters were excluded in the logistic regression because that these data were absent in more than 10% of study subjects.

**Table 1:** Baseline characteristics of the patients and univariate comparisons in derivation cohort

Variable	All patients (*n* = 4252)	Non-AKI (*n* = 3767)	AKI (*n* = 485)	*p* value
Demographic data				
Male, *n* (%)	3414 (80.3)	3042 (80.8)	372 (76.7)	0.035
Age, (years)	58.2 ± 11.6	57.8 ± 11.5	61.4 ± 12.5	< 0.001
Medical history				
Hypertension, *n* (%)	2422 (57)	2094 (55.6)	328 (67.6)	< 0.001
Diabetes mellitus, *n* (%)	1236 (29.1)	1062 (28.2)	174 (35.9)	< 0.001
CVD, *n* (%)	1092 (25.7)	948 (25.2)	144 (29.7)	0.030
Atrial fibrillation, *n* (%)	109 (2.6)	91 (2.4)	18 (3.7)	0.089
CKD, *n* (%)	144 (3.4)	71 (1.9)	73 (15.1)	< 0.001
Hyperlipemia, *n* (%)	1287 (30.3)	1166 (31)	121 (24.9)	0.007
Cerebral infarction, *n* (%)	413 (9.7)	344 (9.1)	69 (14.2)	< 0.001
Previous PCI, *n* (%)	508 (11.9)	436 (11.6)	72 (14.8)	0.037
Clinical data				
Extensive anterior MI, *n* (%)	700 (16.5)	584 (15.5)	116 (24)	< 0.001
STEMI, n (%)	3251 (76.5)	2856 (75.8)	395 (81.4)	0.006
Killip class ≥ 3	426 (10.0)	255 (6.8)	171 (35.3)	< 0.001
Time from AMI attack on admission, (h)	6 (3–14)	6 (3–14)	6.5 (3–14)	0.656
Time from AMI attack to reperfusion, (h)	6 (4–10)	6 (4–9.5)	6 (4–11)	0.097
Time from admission to reperfusion > 120 min, *n* (%)	2196 (51.6)	1916 (50.9)	280 (57.7)	0.004
Coronary angiography, *n* (%)	3884 (91.3)	3497 (92.9)	387 (79.8)	< 0.001
Primary PCI, *n* (%)	2374 (55.8)	2110 (56)	264 (54.5)	0.532
Left main artery, n (%)	254 (6.0)	210 (6.0)	44 (11.4)	< 0.001
Two or more culprit lesions, *n* (%)	2266 (53.3)	2029 (57.8)	237 (61.4)	0.171
Ventricular fibrillation, *n* (%)	137 (3.2)	91 (2.4)	46 (9.5)	< 0.001
3 degree atrioventricular block, *n* (%)	57 (1.3)	34 (0.9)	23 (4.7)	< 0.001
Cardiac arrest, *n* (%)	138 (3.2)	91 (2.4)	47 (9.7)	< 0.001
Shock during hospitalization, *n* (%)	366 (8.6)	202 (5.4)	164 (33.8)	< 0.001
Heart rate > 100 bpm on admission	243 (5.7)	171 (4.5)	72 (14.8)	< 0.001
Systolic BP on admission, (mmHg)	120.5 ± 19.4	120.9 ± 18.9	117.9 ± 22.7	0.006
Diastolic BP on admission, (mmHg)	74.0 ± 11.9	74.2 ± 11.7	73.0 ± 13.3	0.057
echocardiography data				
Initial LVEF on admission, (%)	54.6 ± 9.8	55.1 ± 9.5	50.7 ± 11.1	< 0.001
Initial LVDd on admission, (mm)	49.3 ± 5.4	49.2 ± 5.3	49.9 ± 6.2	0.051
Initial RVDd on admission, (mm)	21.0 ± 5.5	21.0 ± 5.6	21.0 ± 3.8	0.537
E/A > 1 on admission, n (%)	1263 (38.4)	1123 (38.7)	140 (36.6)	0.442
Laboratory data				
Serum creatinine on admission, (umol/L)	74.9 (64.4–88.1)	73.8 (64.0–85.7)	89.0 (70.1–115.8)	< 0.001
eGFR on admission, [ml/(min·1.73 m^2^)]	100.6 (82.6–121.6)	102.3 (85.4–122.7)	80.0 (56.0–105.6)	< 0.001
hCRP on admission, (mg/L)	7.8 (3.0–19.4)	7.3 (2.8–18.0)	11.5 (4.5–30.5)	< 0.001
FBG on admission, (mmol/L)	6.0 (5.4–7.4)	6.0 (5.3–7.3)	6.5 (5.6–8.4)	< 0.001
HBA1C, (%)	6.5 ± 1.5	6.5 ± 1.5	6.6 ± 1.4	0.035
Serum sodium on admission, (mmol/L)	139.1 ± 3.6	139.2 ± 3.5	138.3 ± 4.0	< 0.001
Serum calcium on admission, (mmol/L)	2.2 ± 0.1	2.2 ± 0.1	2.2 ± 0.2	< 0.001
Albumin on admission, (g/L)	39.2 ± 4.1	39.3 ± 3.9	37.9 ± 5.0	< 0.001
Uric acid on admission, (umol/L)	328.9 (270.9–394.9)	326.8 (268.7–390.4)	357.0 (283.8–432.2)	< 0.001
Totalcholesterol, (mmol/L)	4.6 ± 1.1	4.6 ± 1.1	4.5 ± 1.1	0.175
Triglyceride, (mmol/L)	1.5 (1.1–2.2)	1.5 (1.1–2.2)	1.4 (1.0–2.0)	0.158
Low density lipoprotein, (mmol/L)	2.9 ± 0.9	2.9 ± 0.9	2.8 ± 0.9	0.008
High density lipoprotein, (mmol/L)	1.0 ± 0.3	1.0 ± 0.2	1.1 ± 0.3	0.102
Leukocyte on admission,(× 10^9^/L)	10.2 ± 3.6	10.0 ± 3.5	11.2 ± 4.2	< 0.001
Hemoglobin on admission, (g/L)	143.4 ± 17.2	144.3 ± 16.5	137.3 ± 20.7	< 0.001
Hematocrit on admission, (%)	41.5 ± 4.5	41.7 ± 4.3	40.1 ± 5.4	< 0.001
Peak serum TNI ≥ 100 ng/ml, *n*(%)	802 (18.7)	644 (17.1)	158 (32.6)	< 0.001
Peak serum MB, (ug/L)	112.3 (34.0–265.3)	107.5 (32.2–257.2)	173.4 (53.1–299.0)	< 0.001
NT-ProBNP on admission, (pg/mL)	115 (41–327)	104 (39–259)	378 (89–1035)	< 0.001
Treatment				
Furosemide dosage ≥ 60 mg/d, *n* (%)	102 (2.4)	47 (1.2)	55 (11.3)	< 0.001
Intravenous nitrates, n (%)	1689 (39.7)	1442 (38.3)	249 (50.9)	< 0.001
β-blocker, *n* (%)	3253 (76.5)	2905 (77.1)	348 (71.9)	0.011
ACEI/ARB, n (%)	2543 (59.8)	2269 (60.2)	274 (56.5)	0.114
Intravenous thrombolysis, *n* (%)	212 (5.0)	171 (4.5)	41 (8.5)	< 0.001
Use of IABP, *n* (%)	174 (4.1)	107 (2.8)	67 (13.8)	< 0.001
Pulmonary mechanical Ventilation, *n* (%)	192 (4.5)	127 (3.4)	66 (13.6)	< 0.001
Temporary pacemaker, *n* (%)	47 (1.1)	31 (10.8)	16 (3.3)	< 0.001
Contrast volume, (mL)	185.5 ± 102.0	189.7 ± 101.0	153.2 ± 104.0	< 0.001

*CVD* cardiovascular disease, *CKD* chronic kidney disease, *PCI* percutaneous coronary intervention, *AMI* acute myocardial infarction, *BP* blood pressure, *LVEF* left ventricular ejection fraction, *LVDd* left ventricular end-diastolic dimension, *RVDd* right ventricular end-diastolic dimension, *eGFR* estimation of glomerular filtration rate, *hsCRP* high sensitivity C-reactive protein, *FBG* fast blood glucose, *HBA1C* glycosylated hemoglobin, *TNI* troponin I, *CK-MB* creatine kinase isoenzyme, *NT-ProBNP* N-terminal pro-B-type natriuretic peptide, *ACEI* angiotensin converting enzyme inhibitor, *ARB* angiotensin receptor blocker, *IABP* intra-aortic balloon pump

### Multivariable analysis and derivation of prediction score

The results of multivariable logistic regression analysis of backward stepwise variable selection in 4025 patients (representing 94.7% of the derivation cohort) are shown in Table 2. The independent risk factors and prediction score for AKI were as follows: risk score 1 point included hypertension history [OR 1.45, 95% confidence interval (CI): 1.15–1.84], heart rate > 100 bpm on admission (OR 1.75, 95% CI: 1.20–2.55), peak troponin I ≥ 100 μg/L (OR 1.74, 95% CI: 1.34–2.26), and time from admission to coronary reperfusion > 120 min (OR 1.36, 95% CI: 1.08–1.72); risks score 2 points included killip classification [28] ≥class 3 during admission (OR 1.99, 95% CI: 1.45–2.75) and maximum dosage of intravenous furosemide ≥60 mg/d (OR 2.94, 95% CI 1.74–4.99); risks score 3 points only included shock during hospitalization (OR 3.81, 95% CI 2.75–5.28). In addition, when baseline eGFR was less than 90 ml/min·1.73 m^2^, every 10 ml/min·1.73 m^2^ reduction of eGFR (OR 1.52, 95%CI 1.43–1.62) increased risk score 1 point (Tables 2 and 3).

**Table 2:** Multivariate logistic regression in derivation cohort

Variable	β	Odds ratio (95% CI)	*p* value
History of hypertension	0.372	1.45 (1.15–1.84)	0.002
Killip classification ≥ class 3	0.694	1.99(1.45–2.75)	< 0.001
Shock during hospitalization	1.344	3.81 (2.75–5.28)	< 0.001
Every 10 ml/(min. 1.73 m^2^) decline of eGFR under 90 ml/(min. 1.73 m^2^)	0.422	1.52 (1.43–1.62)	< 0.001
HR > 100 bpm at admission	0.564	1.75 (1.20–2.55)	0.004
Peak serum troponin ≥ 100 ng/mL	0.552	1.74 (1.34–2.26)	< 0.001
Time to reperfusion > 120 min	0.312	1.36 (1.08–1.72)	0.010
Intravenous furosemide ≥ 60 mg/d	1.082	2.94 (1.74–4.99)	< 0.001

*eGFR* estimation of glomerular filtration rate, *HR* heart rate

**Table 3:** Prediction score forAKI

Risk factor	risk score
History of hypertension	1
Killip classification ≥ class 3	2
Shock during hospitalization	3
HR > 100 bpm on admission	1
eGFR [ml/(min•1.73 m^2^)] on admission	
80–89.9	1
70–79.9	2
60–69.9	3
50–59.9	4
40–49.9	5
30–39.9	6
≤ 29.9	7
Peak serum troponin ≥ 100 ng/mL	1
Time to reperfusion > 120 min	1
Intravenous furosemide ≥ 60 mg/d	2

*eGFR* estimation of glomerular filtration rate, *HR* heart rate

The prediction score included 8 variables that ranged from 0 to 18 points. Furthermore, patients were categorized into 4 risk groups based on the scores: low risk (0–3 points, 4.8% incidence of AKI), intermediate risk (4–7 points, 13.4% incidence of AKI); high risk (8–11 points, 46.7% incidence of AKI), and very high risk (≥12 points, 81.2% incidence of AKI)(Table 4). To determine the optimal threshold value for predicting AKI, Youden index was used, and the best cut-off in the present model was 3 points (with a sensitivity of 71.1% and specificity 74.2%). The incidence of AKI was significantly higher in patients with scores > 3 points than those with scores ≤3 points (23.0% vs. 4.8%, *P* < 0.001).

**Table 4:** Incidence of acute kidney injury according to prediction score

Score risk category	score	total patients (n)	AKI (n, %)	Death (n, %)
Low	0–3	2711	130 (4.8)	9 (0.3)
Intermediate	4–7	1184	159 (13.4)	17 (2.0)
High	8–11	272	127 (46.7)	25 (12.1)
Very high	≥12	85	69 (81.2)	18 (31.0)

The score ranged from 0 to 18 points

**Fig. 1:**
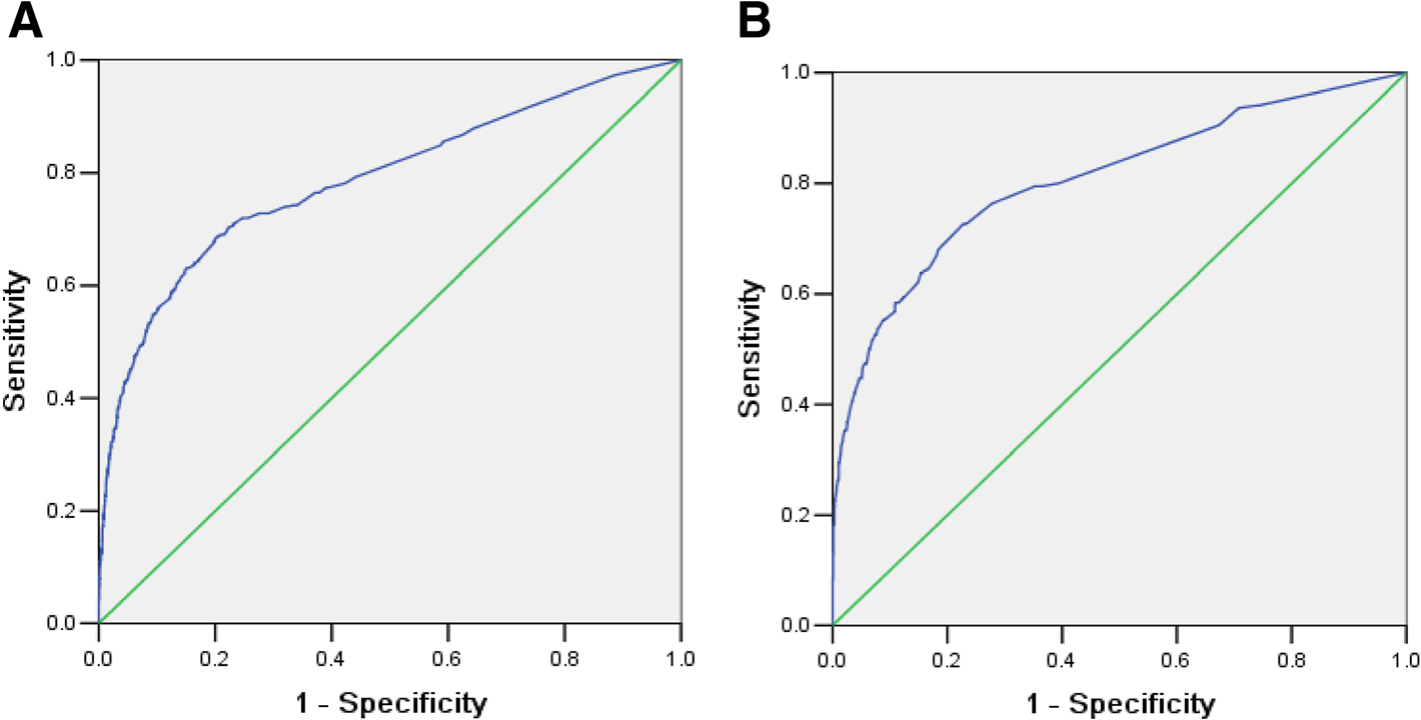
Area under the ROC curve for the derivation and validation sets. **a** Derivation sets, area under the ROC curve 0.79(0.76–0.81). **b** Validation sets, area under the ROC curve 0.81(0.77–0.85)

**Fig. 2:**
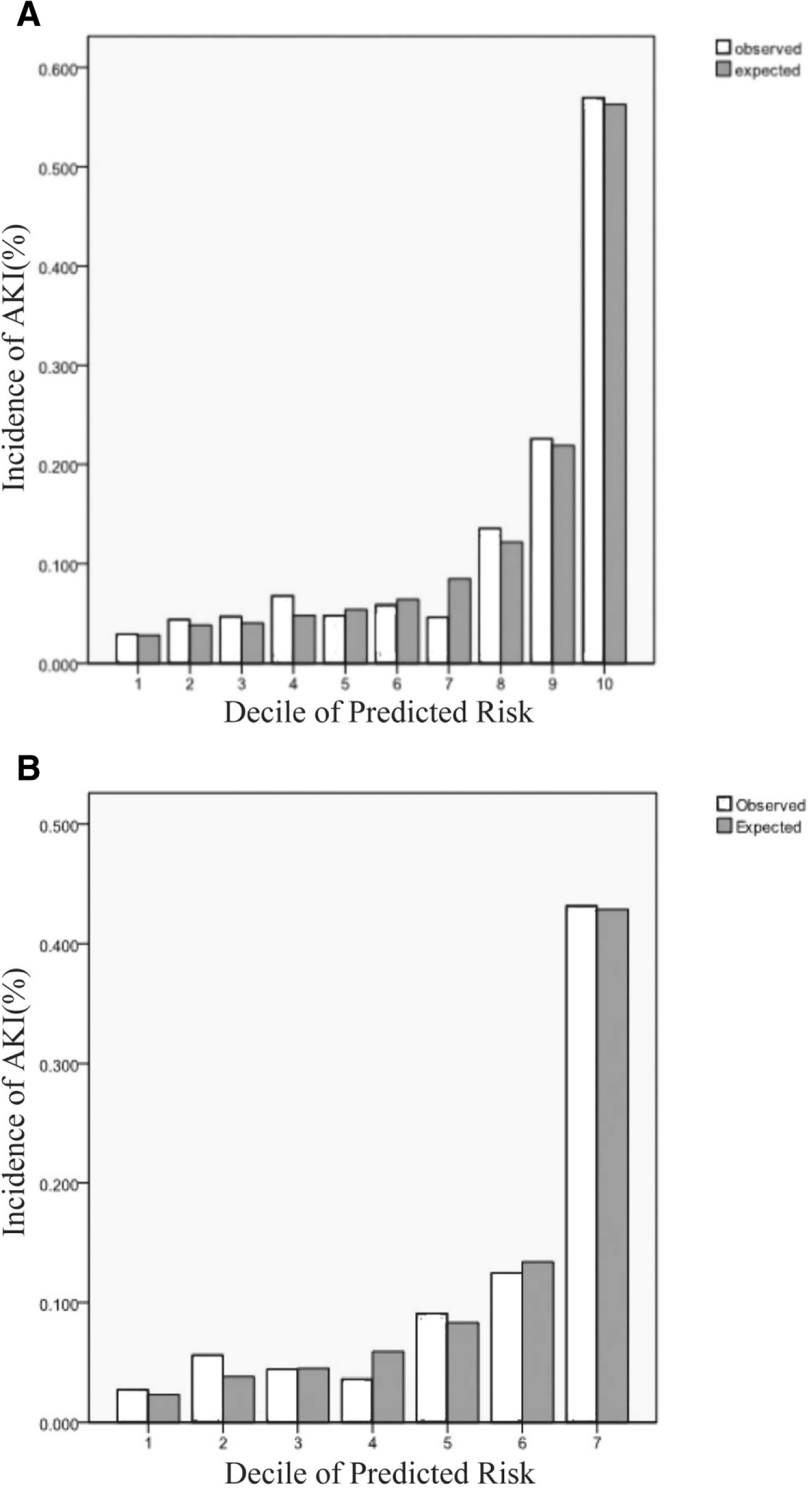
Obseved versus predicted incidence of AKI In derivation and validation sets. **a** Derivation sets, the Hosmer-Lemeshowsatistic χ^2^ = 6.19, *P* = 0.63. **b** Validation sets, the Hosmer-Lemeshowsatistic χ^2^ = 3.64, *P* = 0.60

The prediction score displayed adequate discrimination between patients with or without AKI (AUC: 0.79, 95% CI 0.76–0.81) (Fig. 1a). It was well calibrated across deciles of observed and expected risks of AKI (Hosmer–Lemeshow chi-square value was 6.19, *P* = 0.63) (Fig. 2a).

### Validation of prediction score

An adequate discriminative power was also demonstrated in the validation cohort with an AUC of 0.81 (95% CI: 0.77–0.85) (Fig. 1b). The result of Hosmer-Lemeshow test in the validation cohort was not statistically significant with Chi-square value 3.64, *P* = 0.60 (Fig. 2b).

### External validation of Abusaoda’s prediction score

The prediction score established by Abusaoda based on the definition of AKIN, and it consisted of seven prediction factors including cardiac arrest, decompensated heart failure at admission, diabetes mellitus, hypertension, anemia, impaired renal function at admission, and tachycardia at admission. Because the population of this study was similar to our study and the seven prediction factors in Abusaoda’s prediction scores were all involved in our study, the external validation of this prediction score was performed by us in our total 6014 patients, and the AUC was 0.73 (95% CI: 0.71–0.75). While, using our prediction score in the same group of patients, the AUC was 0.78 (95% CI: 0.76–0.80, *P* < 0.05).

## Discussion

AKI is one of the major complication in AMI patients. The incidence of AKI was reported to be 8.7–36.6% in AMI patients due to the differences in the subjects and the diagnostic criteria [1–8]. In our study, the incidence of AKI in patients with AMI was 11.2%, which was within the scope of the previous literature reports [1–8]. The hospital mortality induced by AKI was also higher: 9.2–39.6% [1, 2, 29, 30]. In the present study, the hospital mortality increased significantly in patients with AKI compared with those without AKI (10% vs. 1.6%, *P* < 0.05), and the length of hospital stay obviously prolonged. In ACTION registry, the in-hospital mortality of patients with AKI was 15%, which was 7.5-fold higher than those without AKI (2%) [1]. Moreover, the occurrence of AKI also affected the long-term prognosis in AMI patients and reduced the long-term survival rate [11, 31]. Therefore, currently, reducing the incidence and mortality of AKI in patients with AMI should be solved urgently. Establishment of the prediction score would provide the foundation for preventing AKI.

A majority of the published studies showed that basal renal dysfunction was the major risk factor for AKI [11, 22, 23, 32]. Our study confirmed this also. The risk of AKI increased 1.52-fold when the baseline eGFR decreased by per 10 mL/min · 1.73 m^2^. Patients with baseline renal dysfunction may be with poor renal reserve function as well as low compensatory ability [33]. After the occurrence of AMI, these patients will be suffered from heart and kidney hypoperfusion and strong stress response, thereby their renal function will be damaged heavily. Previous studies have shown that elderly patients who always have a poor renal reserve capacity was a risk factor for AKI [18–21, 23]. However, the same results were not obtained in this study, which may be related to the age factor being revised when the modified MDRD formala was used to eGFR. In our study, we also found that hypertension was an independent predictor of AKI in patients with AMI, which was consistent with the results of the previous study [1, 22]. Patients with continuous hypertension may result in renal arteriolosclerosis, which leading to chronic renal injury and basal renal dysfunction [34, 35].

In our study, the risk of AKI in patients with killip classification ≥ class 3 during admission was 1.99-fold higher than those with killip classification < class 3. In a retrospective analysis of the data from 2798 patients with AMI, Kuji et al. found that the incidence of AKI in killip 1, killip 2–3, and killip 4 patients were 6.7, 15.3, and 31.3%, respectively [36]. Also, with the worsening of cardiac function, the incidence of AKI increased gradually. Another retrospective study based on the data of 5244 patients with AMI showed that killip 3 or 4 was an independent risk factor for AKI [13], which was consistent with our conclusion. In addition, the risk of AKI in patients with shock was 3.81-fold higher than those without shock, which is similar to other study previously [1]. Finally, we also found that the risk of AKI in patients with troponin I ≥ 100 μg/L was 1.74-fold higher than those patients with troponin I < 100 μg/L. This might be because that troponin I ≥ 100 μg/L were closely related with the occurrence of cardiogenic shock and heart failure [37]. All these three factors can reduce cardiac output, then lead to the decline of renal perfusion as well as renal ischemia, result in AKI ultimately. Recent studies found that patients of heart failure with lower left ventricular ejection fraction, could result in insufficient renal perfusion because of reduced cardiac output. Moreover, the increase of peripheral venous pressure and intraabdominal pressure caused by right cardiac insufficiency can reduce the effective blood flow of the kidneys and activate the inflammatory factors, then caused AKI similarly [38–42].

In the previous two prediction scores created by Queiroz and Abusaada, tachycardia was an independent risk factor for AKI and was included in the prediction score [22, 23]. The similar conclusion was drawn from our study: we found the risk of AKI increased by 1.75 times when heart rate > 100 bpm at admission. This might be attributed to that these patients with tachycardia always had a poor heart function which might result in acute reduction of cardiac output and poorer renal perfusion [43].

The time from admission to coronary reperfusion is one powerful prognostic marker of AKI in patients with STEMI, and also which is a key point to improving the survival after STEMI through shorting the total ischemic duration [44, 45]. Other studies have shown that the time to coronary reperfusion is an independent risk factor for the development of AKI in patients with STEMI [16]. Shacham et al. retrospectively analyzed the data from 417 patients of STEMI. The incidence of AKI according to the time to reperfusion was 6.6% with < 120 min, 9.7% with 120–300 min, and 13.3% with > 300 min. After multivariable regression correction, time to coronary reperfusion still as an independent predictor of AKI [16]. Our study showed a similar conclusion that the time more than 120 min from admission to coronary reperfusion was an independent risk factor for AKI in patients with AMI. The sudden myocardial insult of AMI often results in an acute reduction of cardiac output and renal perfusion. Early short-time of hemodynamic deterioration only cause a reversible loss of renal function without structural damage of kidney. However, prolonged renal hypoperfusion would lead to acute tubular necrosis ultimately [46]. Therefore, timely recovery of coronary artery perfusion can solve hemodynamic instability, improve left ventricular ejection fraction and solve arrhythmia as well other problems, so as to resume renal perfusion and reduce the incidence of AKI finally [16].

In the present study, we have confirmed that larger dosage of intravenous loop diuretics were the cause of AKI. The risk of AKI in patients with intravenous furosemide dosage ≥60 mg/d was 2.9-fold higher than those patients with < 60 mg/d. We analyzed the data of 1010 patients with acute heart failure and acute exacerbation of chronic heart failure, and found that the risk of AKI in patients with intravenous furosemide dosage ≥80 mg/d and ≥ 120 mg/d was nearly 1.96- and 5.06-fold higher than those with < 80 mg/d [47]. Although Use of loop diuretics can reduce venous congestion and increase renal blood flow, larger dosage might also reduce circulating blood volume, decrease the renal blood flow, activate the sympathetic and renin-angiotensin system, and increase the peripheral vascular resistance, thereby lead to a decreased renal function [48]. Therefore, the use of diuretics is a double-edged sword, and inappropriate use of larger dosage can lead to renal damage.

Our study did not found the significant relation between contrast volume and AKI, which was consistent with the results of another study [18]. We found the incidence of contrast-induced nephropathy (CIN) in our hospital was only 4.5% (177/3945) [21]. Therefore, we speculate that CIN is no longer a major risk factor for AKI in patients with AMI due to the widely use of isotonic contrast agent and the gradually enhancement of preoperative hydration awareness of cardiologists. So the patients who undergone multiple PCI (i.e., multiple use of contrast agents) were not be excluded in the present study.

Some studies have proposed prediction scores for AKI in the patients with AMI, but most of them were to assess the risk of CIN after PCI or coronary angiography [18–21]. AMI itself also causes deleterious haemodynamic, immunologic and neuroendocrine effects on kidney function except the effects of contrast medium. Moreover, outcome of some AMI patients was not treated with PCI or coronary angiography. Therefore, it is important to create prediction scores for AKI including all the AMI patients which undergoing PCI or not. Presently, two studies have developed prediction scores for AKI in this part of the patients with AMI [22, 23]. Compared with these two prediction scores, our study has displayed some characteristics as following: Firstly, the prediction score of Queiroz are mainly applicable to identify the risk of AKI in the emergency patients with STEMI [22]. The prediction score from Abusaada can predict the early risk of AKI only in AMI patients who have just been hospitalized [23]. The current prediction score in our study analyzed the risk factors of AKI on admission as well as some possible risk factors of AKI during hospitalization. Therefore it can evaluate the occurrence of AKI in patients with AMI more comprehensively. The prediction score of Abusaada was validated by us based on the data from 6014 patients in our study. The results show that the AUC in the prediction score from Abusaada was lower (0.73) than that in our prediction score (0.78, *P* < 0.05), which might be attributed to that the prediction score in our study simultaneously assessed the risk of AKI not only during admission but also within hospitalization. Therefore, we think that our prediction score is more valuable to predict the occurrence of AKI. And it also suggests that if there are more risk factors for AKI when patients admitted to hospital, we need to pay more attention to avoiding the side effect of treatment and drugs on the kidney after admission. Secondly, our prediction score have been involved in the time from admission to coronary reperfusion and larger dosage of intravenous loop diuretics, and all of these two were modifiable risk factors, which also suggested that some AKI after AMI can be avoided. We should shorten the time from admission to reperfusion and avoid the use of larger dosage of intravenous loop diuretics. All of these can prevent the occurrence of AKI in patients with insufficient basal renal reserve and hemodynamic changes. Finally, the two prediction scores created by Queiroz and Abusaada were without validation. we not only randomly selected 70% patients as derivation cohort but also 30% patients as validation cohort, and the AUC were 0.79 and 0.81, respectively; while the Hosmer-Lemeshow *P-*values were 0.63 and 0.60.

The prediction score is easy to be calculated and also has a certain clinical practicality. With eight common clinical variables, the prediction score is relatively simple to calculate. If we use this prediction score in AMI patient, then we can get the corresponding AKI risk score. It is very helpful for clinicians to have a preliminary judgment on the risk of the AMI patients belongs to. High risk and very high risk patients may be required with frequents monitoring, preventive strategies, and even with priority treatment, in order to be with a well renal outcome finally.

Although our prediction score is based on large data, however there must be some limitations with it because that is a retrospective analysis from a single center, so its inherent weakness cannot be avoided. The more accurate incidence of AKI described in our study might be underestimated, which because some patients might already have kidney injury before presentation although we used Scr level on admission to calculate baseline renal function.

## Conclusion

We have developed a validated prediction score to predict AKI in patients with AMI. The application of such a predictive tool may help clinicians to have a preliminary judgment on the AKI risk of the AMI patients. Hence frequents monitoring, preventive strategies, and even with priority treatment should be given to the high risk patients, in order to be with a well renal outcome finally in AMI patients.

## Abbreviations

AKI: acute kidney injury
AKIN: acute kidney injury network
AMI: acute myocardial infarction
AUC: area under the receiver operating characteristic curve
CI: confidence interval
eGFR: estimated glomerular filtration rate
MDRD: modification of Diet in Renal Diseases
OR: odds ratio
ROC: receiver operating characteristic
SCr: serum creatinine
STEMI: ST-segment elevation myocardial infarction
CIN: contrast-induced nephropathy
